# *Lactobacillus johnsonii* glycolipids, their structure and immunoreactivity with sera from inflammatory bowel disease patients

**DOI:** 10.1111/1751-7915.12424

**Published:** 2016-10-21

**Authors:** Mariola Paściak, Sabina Górska, Natalia Jawiarczyk, Andrzej Gamian

**Affiliations:** ^1^ Hirszfeld Institute of Immunology and Experimental TherapyPolish Academy of SciencesRudolfa Weigla 1253‐114WrocławPoland

## Abstract

Structural studies of the major glycolipids produced by two *Lactobacillus johnsonii* (LJ) strains, LJ 151 isolated from intestinal tract of healthy mice and LJ 142 isolated from mice with experimentally induced inflammatory bowel disease (IBD), were performed. Two major glycolipids, GL1 and GL2, were present in lipid extracts from *L. johnsonii* 142 and 151 strains. Glycolipid GL1 has been identified as β‐D‐Glc*p*‐(1→6)‐α‐D‐Gal*p*‐(1→2)‐α‐D‐Glc*p*‐diglyceride and GL2 as α‐D‐Gal*p*‐(1→2)‐α‐D‐Glc*p*‐diglyceride. The main fatty acid residues identified by gas‐liquid chromatography–mass spectrometry were palmitic, stearic and lactobacillic acids. Besides structural elucidation of the major glycolipids, the aim of this study was to determine the immunochemical properties of these glycolipids and to compare their immunoreactivity to that of polysaccharides obtained from the same strains. Sera from rabbits immunized with bacterial cells possessed much higher serological reactivity with polysaccharides than with glycolipids. Inversely, reactivity of the glycolipids with human sera from patients with IBD was much higher than that determined for the polysaccharides, while reactivity of glycolipids with human sera from healthy individuals was much lower than one measured for the polysaccharides. Results indicate that glycoconjugates from *Lactobacillus* cell wall act as antigens and may represent new IBD diagnostic biomarkers.

## Introduction

Bacteria of the *Lactobacillus* genus are commonly found in fermented food products. They are important in food production that require lactic acid fermentation, in particular dairy products, vegetables, meat and sourdough bread. Lactobacilli are also a major colonizer of the mucosa of the human and animal's gastrointestinal tract. Bacteria of the *Lactobacillus* genus exert a number of beneficial effects including modulation of the immune system activity of the host, improved healing of damaged gastric and intestinal mucosa, reducing lactose intolerance or inducing hypocholesterolaemic action, protection against microbial infection and maintenance of the homeostasis in the intestine (Oelschlaeger, 2010). It is well established that *Lactobacillus* bacteria can be used in prevention and treatment of such intestinal inflammatory disease as inflammatory bowel disease (IBD) (Sheil *et al*., 2007; Orel and Kamhi Trop, 2014; Polito, 2014; Shen *et al*., 2014; Saez‐Lara *et al*., 2015; Shadnoush *et al*., 2015). IBD is a term covering two diseases characterized by inflammatory cell infiltration to intestinal submucosa: ulcerative colitis and Crohn's disease. The aetiology of IBD is largely unknown but it is generally accepted that the chronic inflammation is perpetuated by intestinal commensal microbiota in genetically predisposed hosts with altered immune response to bacterial products (Young and Abreu, 2006). However, the precise molecular mechanisms by which these bacterial cell wall‐associated molecules exert their effects in the intestine have not yet been clearly elucidated. It is possible that the body's own tissue causes an autoimmune response. Uncontrolled inflammatory reaction during the IBD (regardless of what is the cause of the inflammation) leads to damage of intestinal wall and cause diarrhoea and pain.

The dysbiosis of intestine microbiota and reduction in microbial diversity has been observed in IBD (Sartor, 2008). There is an increasing evidence that the reduction of mucosa‐associated *Bifidobacterium* spp. or *Lactobacillus* spp., belonging to commensal microbiota, along with an increased relative abundance in pathogenic bacteria is associated with IBD pathogenesis (Ott *et al*., 2004; Strober *et al*., 2007; Khor *et al*., 2011).

*Lactobacillus johnsonii* (LJ) is one of the many species typically found in human and animal gastrointestinal tract as a part of normal, commensal microbiota and shown to be a beneficial microorganism (Falsen *et al*., 1999; La Ragione *et al*., 2004; Kaburagi *et al*., 2007). Over the past few years, the use of different *Lactobacillus* species in IBD treatment has gained attention. Manipulation of the colonic bacteria with beneficial agents may prove to be more effective and may have the potential to open new possibilities in prevention or treatment these diseases. Few clinical trials were conducted with LJ La1 strain but no sufficient effect was observed (Marteau *et al*., 2006; Van Gossum *et al*., 2007).

Cell wall glycoconjugates of lactobacilli are studied for their antigenic properties and for potentially health‐promoting interactions with the host (Wells, 2011). These glycoconjugates are chiefly comprised of polysaccharides (Kleerebezem *et al*., 2010), glycoproteins (Kleerebezem *et al*., 2010) and glycolipids (Shaw, 1970; Iwamori *et al*., 2011). Polysaccharides of lactobacilli are mainly heteropolysaccharides with different structures, modes of linkage and substitutions. They are thought to play a role in protecting lactobacilli against desiccation, toxic compounds, bacteriophages, osmotic stress and permitting adhesion to solid surfaces (De Vuyst and Degeest, 1999). Such adhesion is responsible for biofilm formation. Recently, polysaccharides’ role in host–microbe interactions (Conover *et al*., 2010) and in immunomodulation (Lebeer *et al*., 2010; Górska *et al*., 2014) has been reported. In our previous studies, we have shown the presence of structurally and immunologically distinct polysaccharides derived from LJ 142 and 151 (Górska *et al*., 2010; Górska‐Frączek *et al*., 2013). Herein, we have established the structure and determined immunoreactivity of major glycolipids from LJ 142 and 151. The LJ 142 strain was isolated from intestine of the mice with experimentally induced IBD, while the 151 strain from intestine of the control mice. Structural studies of glycolipids were initiated in 1970s of the twentieth century (Shaw, 1970), when among glycolipids from Gram‐positive bacteria that of *Lactobacillus* strains had also been investigated. In general, glycolipids of *Lactobacillus* belong to glycoglycerolipids type, composed of glucose and galactose residues, and glycerol substituted with fatty acids including one, characteristic for the whole genus, lactobacillic acid – 11,12‐methyleneoctadecanoic acid (Uchida and Mogi, 1973; Johnsson *et al*., 1995). Depending on the strain, the glycolipid molecule may contain mono, di, tri and tetrasaccharides, and a broad spectrum of the fatty acyl substituents. The most prevalent glycolipids of *Lactobacillus* are diglycosyldiglycerides, α‐D‐Galp‐(1‐2)‐α‐D‐Glcp‐ diglyceride, common in *Lactobacillus* species, found in *L. johnsonii*,* L. reuteri*,* L. fermentum*,* L. rhamnosus*,* L. casei* and *L. plantarum* (Iwamori *et al*., 2011; Sauvageau *et al*., 2012). Three triglycosyldiglycerides structures differing in anomeric configuration of glucose or galactose attached to Gal‐Glc‐diglyceride and/or not substituted with fatty acids were reported, i.e. Galα1‐6Galα1‐2Glcα1‐3'DG in *L. intestinalis* (Iwamori *et al*., 2011); Glcβ1‐6Galα1‐2Glcα1‐3'DG and acylated form Glcβ1‐6Galα1‐2Glc(6‐fatty acid)α1‐3'DG present in *L. casei* (Iwamori *et al*., 2011) and *L. plantarum* (Sauvageau *et al*., 2012). The less common are tetraglycosyldiglycerides, Galα1‐6Galα1‐6Galα1‐2Glcα1‐3'DG reported in *L. intestinalis* and *L. johnsonii* (Iwamori *et al*., 2011).

Bacterial glycolipids possess important function in stabilizing the cell membrane. They may also play a role in strain's virulence (Reed *et al*., 2004) and in formation of the molecular pattern recognized by the host immune system (Cambier *et al*., 2014). Some of *Lactobacillus* cell membrane glycolipids are the lipid anchor for lipoteichoic acids (Jang *et al*., 2011). Besides structural elucidation of the major glycolipids from LJ strains, 151 and 142, the aim of this study was to determine the immunochemical properties of these glycolipids and to compare their immunoreactivity to that of polysaccharides obtained from the same strains.

## Results

### Isolation and purification of glycolipids

*Lactobacillus johnsonii* strains 142 and 151 were cultivated on MRS broth in 37°C for 72 h that corresponded to stationary phase, with yield 10.36 and 6.15 g of wet biomass l^−1^ respectively. TLC analysis of lipid extracts of both *Lactobacillus* strains revealed two major glycolipids labelled GL1 (Rf = 0.55) and GL2 (Rf = 0.66). The TLC spots gave positive reaction with orcinol and vanillin (Mordarska and Paściak, 1994), but there was no staining observed with ninhydrin and the reagent of Dittmer and Lester for phosphorus (Dittmer and Lester, 1964). TLC analysis of crude lipids isolated from *Lactobacillus* representatives, *L. casei*,* L. reuteri*,* L. animalis/murinus* and *L. rhamnosus*, showed that glycolipid GL2 is a major glycolipid of *Lactobacillus* genus (Fig. S1).

The glycolipids of LJ strains 142 and 151 were isolated using column adsorption chromatography and were further purified by HPLC. The column adsorption chromatography on Silica Gel 60 allowed the separation of major glycolipids from neutral lipids and phospholipids. The glycolipid fractions constituted 32–35% of crude lipid. Glycolipid GL1 was purified by column chromatography with a step‐gradient of methanol in chloroform, followed by preparative TLC to remove residual phospholipids and finally by HPLC with methanol–chloroform gradient. Glycolipid GL2 was also separated by column adsorption chromatography on Silica Gel 60, followed by HPLC step. For LJ 151, 1.7 mg of GL1 and 5.1 mg of GL2 were obtained with yield of 0.21% and 0.60% from crude lipid, respectively, while for LJ 142, 1.32 mg of GL1 and 3.78 mg of GL2 were purified with the corresponding yields of 0.17% and 0.47%.

### Chemical analysis

Sugar content, as determined by phenol–sulfuric acid method (Dubois *et al*., 1956), for GL1 was twofold higher than for GL2, i.e. 15.5% for GL1 LJ 151 and 7.5% for GL2 LJ 151 (Table 1). Gas‐liquid chromatography–mass spectrometry (GLC‐MS) analysis of alditol acetates derivatives revealed that both glycolipids possessed glucose, galactose and glycerol, with Glc:Gal molar ratios of 2:1 for GL1 and 1:1 for GL2. Methylation analysis of glycolipid GL1 showed the presence of three components, namely 2,3,4,6‐Me_4_‐Glc (t‐Glc), 3,4,6‐Me_3_‐Glc (2‐Glc), 2,3,4‐Me_3_‐Gal (6‐Gal) at molar ratio approximately 1:1:1. Methylation analysis of glycolipid GL2 revealed the presence of two components namely 2,3,4,6‐Me_4_‐Gal (t‐Gal) and 3,4,6‐Me_3_‐Glc (2‐Glc) at molar ratio 1:1 (Table 1).

**Table 1:** Chemical characteristics of glycolipids G1 and G2 from *L. johnsonii* 142 (LJ 142) and 151 (LJ 151)

	GL1 LJ 142	GL1 LJ 151	GL2 LJ 142	GL2 LJ 151
Rf value	0.55	0.55	0.66	0.66
Yield (%)^a^	0.17	0.21	0.47	0.60
Total sugar (%)^b^	13.8	15.5	7.7	7.50
Glc^c^	1.81	2.10	1.07	0.97
Gal^c^	1.0	1.0	1.0	1.0
t‐Glc^d^		0.87		tr
t‐Gal^d^		tr		0.82
2‐subst Glc^d^		1.0		1.0
6‐subst Gal^d^		0.66		–
Fatty acids (%)^e^				
C_16:0_	45.4	46.6	44.3	53.3
C_18:1_	14.8	15.7	14.3	9.7
C_18:0_	28.9	24.6	18.8	13.9
C_19:0_ cyclo	10.9	13.0	22.5	22.9

a The yield of glycolipid refers to the per cent amount obtained from the crude lipid extract.

b The total neutral sugar in glycolipid determined by the phenol/sulfuric acid method (Dubois *et al*., 1956).

c Molar ratio as determined by sugar analysis with use of GLC‐MS.

d Linkage type and molar ratio as determined by methylation analysis with use of GLC‐MS.

e Fatty acid compositions were determined by GLC of the fatty acid methyl esters.

The main fatty acid residues identified by GLC‐MS were saturated and monounsaturated hexadecanoic, octadecanoic acids and lactobacillic acid with smaller amounts of C14:0, C 16:1 (Table 1).

### NMR analysis

The NMR analysis showed that GL1 glycolipids isolated from LJ 151 and LJ 142 strains have the same structure, also GL2 structure from both strains was identical (Fig. S2). The detailed NMR analysis was performed for GL1 and GL2 isolated from LJ 151.

**Figure 1:**
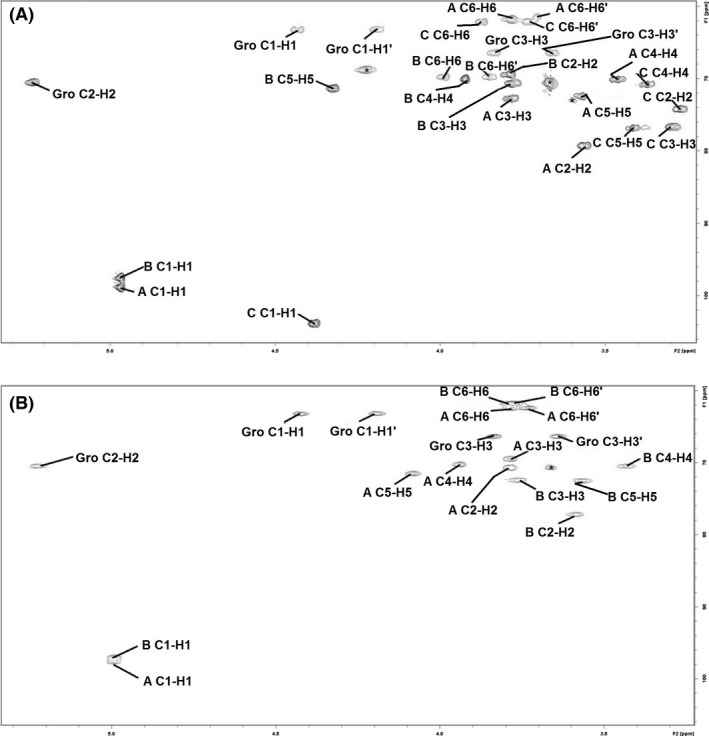
Two dimensional NMR spectra of major glycolipids isolated from *Lactobacillus johnsonii*. Parts of a 2D ^1^H,^13^C HSQC spectra of glycolipids GL1 (A) and GL2 (B) isolated from *L. johnsonii* 151. The spectra were obtained for CDCl_3_/CD_3_ OD (2:1, v/v) solvent at 600 MHz and 22°C. The corresponding parts of the ^1^H and ^13^C NMR spectra are shown along the horizontal and vertical axes respectively. The letters refer to carbohydrate residues and the Arabic numerals refer to proton/carbon in the respective residue denoted as shown in Table 2. The asterisk refers to contamination.

The one‐dimensional ^1^H NMR spectrum at 600 MHz of GL1 LJ 151 contained three anomeric proton signals (A, B, C) at 4.97, 4.96, 4.38 ppm, whereas ^1^H NMR spectrum of GL2 LJ 151 revealed two anomeric proton signals (A, B) at 4.99 and 4.98 ppm. Proton signals correlated with carbon resonances: GL1 at 97.5, 98.8, 103.8 ppm, whereas GL2 at 97.1 and 97.0 ppm, respectively, as shown in the ^1^H‐^13^C HSQC spectrum of GL1 (Fig. 1A) and GL2 (Fig. 1B). The anomeric carbon resonances of all residues are characteristic for pyranose ring form (Altona and Haasnoot, 1980). The ^1^H NMR spectrum of GL1 and GL2 revealed also the presence of the glycerol molecule at δ 4.42/4.19, 5.24, 3.83/3.65 ppm and at 4.43/4.19, 5.22, 3.83/3.64 ppm respectively. The complete ^1^H and ^13^C NMR resonances were obtained using different 2D NMR experiments as well as by comparison with previously published ^1^H and ^13^C NMR data (Gorin and Mazurek, 1975; Bock and Pedersen, 1983; Lipkind *et al*., 1988) and the chemical shifts are reported in Table 2. The ^1^H and ^13^C NMR chemical shifts for H5 and C5, at δ 72.5, 71.3 ppm and at δ 71.5, 72.4 ppm indicated that residues A, B of GL1 and A, B of GL2 respectively, are α‐linked, whereas chemical shifts at 76.7 ppm in C of GL1 indicated that residue C is β‐linked. The sequence of the monosaccharide residues within the repeating unit of the GL1 and GL2 glycolipids and linkage between sugar and glycerol residues was obtained by assignment of the inter‐residue interactions observed in the 2D ROESY and HMBC spectra. A cross‐peak at δ 4.42, 4.19/174.40 ppm in the HMBC spectrum of GL1 identified the linkage of one fatty acid R1 to C‐1 of Gro. Cross‐peaks at δ 4.98/3.83, 3.64 ppm (H1 Glc/H3a; H3b Gro) in the ROESY spectrum of GL2, together with a cross‐peak at δ 3.83, 3.64/102.2 ppm in the HMBC spectrum of GL2, are consistent with the structure Glc‐(1→3)‐Gro. A cross‐peak at δ 4.43, 4.19/174.70 ppm in the HMBC spectrum of GL2 identified the linkage of one fatty acid to C‐1 of Gro. The linkage of the second fatty acid of both glycolipids was indicated to be at C‐2 of Gro due to the great deshielding found for H‐2 (δ 5.22 ppm) and comparison with published similar structures (Paściak *et al*., 2010).

**Table 2:** ^1^H and ^13^C NMR chemical shifts and selected inter‐residue connectivities from the anomeric protons of GL1 and GL2 from *Lactobacillus johnsonii* 151

	Sugar residue	Chemical shifts (ppm)						Connections	
		H‐1	H‐2	H‐3	H‐4	H‐5	H‐6a/H‐6b	HMBC	ROESY
		C‐1	C‐2	C‐3	C‐4	C‐5	C‐6		
GL1									
A	→2)‐α‐D‐Glc*p*‐(1→Gro	4.97	3.56	3.78	3.46	3.57	3.78	A1‐3Gro	A1‐3Gro
		97.5	79.3	72.6	70.0	72.5	61.8		
B	→6)‐α‐D‐Gal*p*‐(1→	4.96	3.80	3.78	3.92	4.32	3.85/3.98	B1‐A2	
		98.8	69.4	70.6	69.9	71.3	69.7		
C	β‐D‐Glc*p*‐(1→	4.38	3.27	3.42	3.37	3.39	3.87/3.73	C1‐B6	
		103.8	74.1	76.8	70.7	76.7	62.1		
Gro		4.42/4.19	5.24	3.83/3.65					
		63.2	70.4	66.4					
GL2									
A	α‐D‐Gal*p*‐(1→	4.99	3.78	3.78	3.94	4.08	3.78/3.74	A1‐B2	A1‐2B
		97.1	69.5	70.7	70.2	71.5	62.4	A1‐A5	
B	→2)‐α‐D‐Glc*p*‐(1→Gro	4.98	3.56	3.76	3.42	3.55	3.69/3.80	B1‐B5	B1‐3Gro
		97.0	77.2	72.3	70.4	72.4	61.8	B1‐3Gro	
Gro		4.43/4.19	5.22	3.83/3.64					
		63.1	70.5	66.3					

Spectra were obtained for CDCl_3_/CD_3_OD (2:1, v/v) solvent at 22°C and the chemical shifts measured relative to chloroform. Arabic numerals refer to protons and carbons in sugar residues denoted by letters. Proton signals were assigned in the COSY, TOCSY, ROESY and HMBC spectra, whereas carbon signals were assigned in the HSQC spectrum. The inter‐residue interactions were observed in the 2D ROESY and HMBC spectra. The ROESY spectra showed inter‐residue rotating frame Overhauser effects (ROEs) between protons, whereas the HMBC spectra showed cross‐peaks between the anomeric proton and the carbon at the linkage position.

The structure of sugar part of the glycolipids GL1 and GL2 is thus as:

$$\begin{array}{cccc} \text{GL}1: & \beta -\text{D‐Glc}p-(1\to 6)\;\;\;\;\;\;\;\;\;\;\;\;\;\;\;\;\;\;\; & -\alpha -\text{D‐Gal}p-(1\to 2) & \;\;\;\;\;\;\;\;\;\;\;-\alpha -\text{D‐Glc}p-(1\to 3)-\text{Gro}\\ & C & B & A\\ \text{GL2}: & \alpha -\text{D‐Gal}p-(1\to 2) & -\alpha -\text{D‐Glc}p-(1\to 3)-\text{Gro}\\ & A & B \end{array}$$

Utilizing ^1^H COSY, TOCSY, HSQC and HMBC characteristic fatty acids, signals of both glycolipids were identified as –CH_2_–CH_2_–COOR (δ 2.33/34.5 ppm), –CH_2_–CH_2_–COOR (δ 1.62/24.6 ppm), CH_2_‐groups (δ 1.25–1.35/∼29.5–31.5 ppm), –CH_3_‐groups (δ 0.89/14.2 ppm), –CH_2_–CH (δ 2.026/27.7 ppm) and ‐CH_2_–CH (δ 5.358/129.9 ppm) for R1 and for R2 were identified as –CH_2_–CH_2_–COOR (δ 2.33/34.5 ppm), –CH_2_–CH_2_–COOR (δ 1.62/24.6 ppm), CH_2_‐groups (δ 1.25–1.35/∼28.5–32.0 ppm), –CH_3_‐groups (δ 0.89/14.2 ppm) and –CH‐groups (δ 0.66/16.0 ppm) (Table S1).

### MALDI‐TOF MS analysis

The both glycolipids were analysed by MALDI‐TOF MS. The mass spectra of glycolipid GL2 from both LJ 142 and 151 were nearly identical with the same *m/z* values for the major molecular ions and similar distribution pattern of the other ions. The peaks ranged from *m/z* 915.513 to 971.503 were predicted to be dihexosyl‐diacyl‐glycerol, possessing two acyl chains ranging from C14 to C22. The most abundant ion at *m/z* = 955.540 was in agreement with a quasi‐molecular ion [M+Na]^+^ consisting of two hexoses, glycerol, lactobacillic and hexadecanoic acyl chains.

The glycolipids GL1 from LJ 142 and 151 were nearly identical with the same m/z values for the major molecular ions and peaks ranging from *m/z* 1077.567 to 1177.596. The mass difference of the quasi‐molecular ion between GL1 and GL2 (Δm = 162 m/z) was in agreement with presence of the additional hexose residue in GL1.

To verify our hypothesis, glycolipid GL1 was subjected to MALDI‐TOF in LIFT mode analysis. The MS/MS analysis was performed on the molecular ion (*m/z* = 1117.793 for [C56H102O20Na]^+^) in positive ion mode. The observed fragmentation spectrum revealed two major ion peaks *m/z* = 821.277 and 861.320 that can be attributed to the loss of lactobacillic acid and palmitic acid respectively. In addition, readily recognizable were signals for the loss of one hexose (Hex) (*m/z* = 954.565), two hexose residues (*m/z* = 793.401) as well as the Hex2 (*m/z* = 346.870) and Hex3 (*m/z* = 508.924) fragments. Other fragments were also identified and listed in Table 3.

**Table 3:** The MALDI LIFT‐TOF/TOF fragment ions of major quasi‐molecular ion of GL1 at *m/z* 1117.793 and GL2 at *m/z* 955.739 of *L. johnsonii* 151 compared with the calculated masses. MALDI‐TOF MS was performed in the positive ion sweep, LIFT mode, using the Ultraflextreme unit (Bruker Daltonics). The glycolipids and norharmane matrix were dissolved in chloroform–MeOH (9:1 v/v)

Measured molecular mass m/z	S/N	Intensity	Chemical formula	Components + Na	Calculated molecular mass
GL1					
346.870	175	2343	C12H21O10Na	2 Hex	348.10
497.086	40	598	C25H46O8Na	Hex+Gro+C16:0	497.30
508.924	80	1228	C18H30O15Na	3 Hex	509.14
566.974	80	1359	C21H36O16Na	3 Hex+Gro	567.19
793.401	191	4634	C44H82O10Na	Hex+Gro+C16:0 + C19:0cycl	793.58
821.277	688	17 299	C37H66O18Na	3 Hex+Gro+C16:0	821.41
861.320	304	7750	C40H70O18Na	3 Hex+Gro+C19:0cycl	861.44
954.565	5	129	C50H92O15Na	2 Hex+Gro+C16:0+C19:0cycl	955.63
GL2					
346.833	28	376	C12H21O10Na	2 Hex	348.10
404.869	25	361	C15H26O11Na	2 Hex + Gro	405.13
497.020	34	534	C25H46O8Na	Hex+Gro+C16:0	497.31
537.052	19	339	C28H50O8Na	Hex+Gro+C19:0cycl	537.34
659.113	316	7638	C31H56O13Na	2Hex+Gro+C16:0	659.36
699.162	140	3637	C34H60O13Na	2Hex+Gro+C19:0cycl	699.39
793.431	171	4947	C44H82O10Na	Hex+Gro+C16:0+C19:0cycl	793.58

The MALDI LIFT‐TOF/TOF mass spectrum obtained for the molecular ion *m/z* = 955.739 for [C50H92O15Na]^+^ of glycolipid GL2, in positive ion mode revealed two major fragment ion peaks *m/z* = 659.113 and 699.162 that can be attributed to the loss of lactobacillic acid and palmitic acid respectively. The other ions, e.g. after loss of one hexose (*m/z* = 793.431), the loss of Hex and either one of the two fatty acids (*m/z* = 497.020 and *m/z* = 537.052) were also identified (Table 3).

### Immunoreactivity of glycolipids and polysaccharides with non‐immune mouse and rabbit sera

Immunological activity of glycoconjugates isolated from *L. johnsonii* (LJ142 and LJ151), both glycolipids and polysaccharides (Górska *et al*., 2010; Górska‐Frączek *et al*., 2013), was tested by enzyme‐linked immunosorbent assay (ELISA) using sera from mice kept under specific pathogen‐free conditions and also with non‐immune rabbits. Pure polysaccharides LJ 151 and LJ 142 reacted only with non‐immune mouse sera; however, the reactivity of PS 151 was 40% higher than of PS 142. In the case of the glycolipids, neither GL1 nor GL2 showed reactivity with tested sera (Fig. S3). As we have shown pre‐immune rabbits sera have no reactivity with polysaccharides and glycolipids of *L. johnsonii*. This observation leads us to use the rabbit model for obtaining polyclonal antibodies.

### Immunoreactivity of glycolipids and polysaccharides with rabbit sera

The rabbit antisera raised against LJ 151 and 142 whole cells were obtained and used in passive hemagglutination assay and tested by immunodiffusion using protein antigens (cell homogenates) of *L. johnsonii*. In rabbit sera, passive hemagglutination assay showed antibodies raised against LJ 151 and 142 cells at 1/512 and 1/384 titres respectively. The presence of the above‐described antibodies was confirmed by the double immunodiffusion test (data not shown).

**Figure 2:**
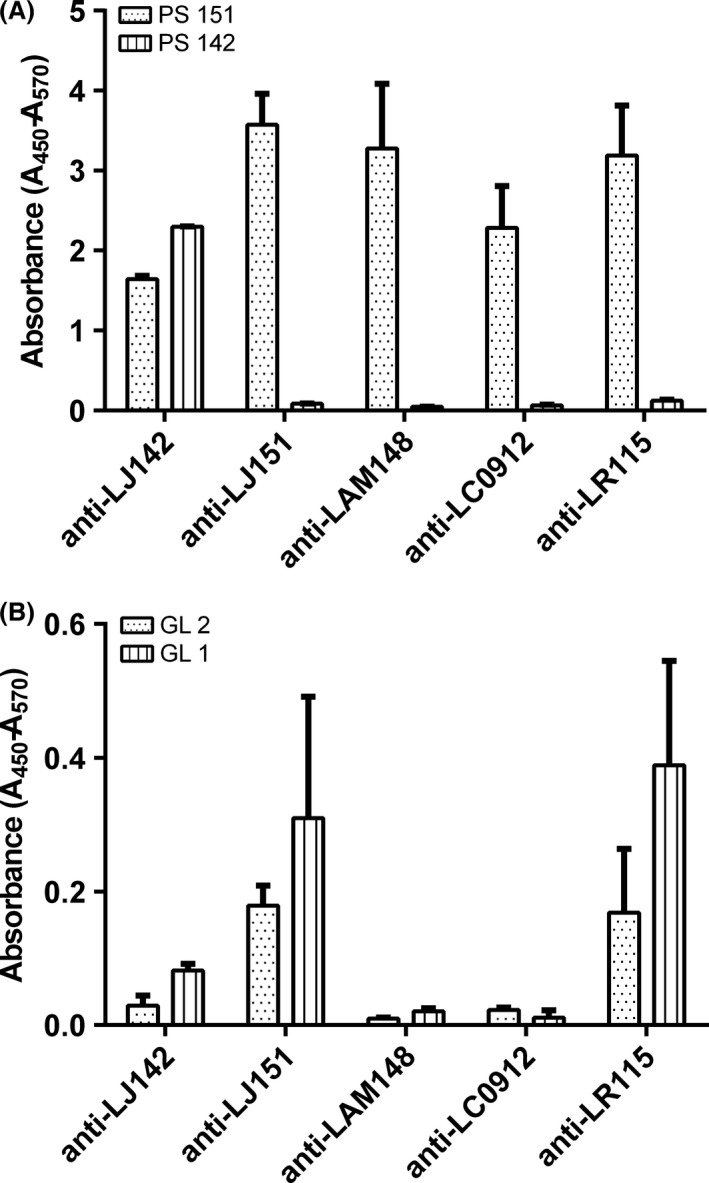
Reactivity in ELISA of *L. johnsonii* polysaccharides and glycolipids with rabbit polyclonal sera. The plate was coated with polysaccharides (PS 142, PS 151) (A) and glycolipids (GL1, GL2) (B) from *L. johnsonii* strains 151 and 142. Rabbit sera anti cell mass of *L. johnsonii* 142 (LJ142), *L. johnsonii* 151 (LJ151), *L. animalis*/*murinus* 148 (LAM148), *L. casei* 0912 (LC0912) and *L. reuteri* 115 (LR115) were used as the primary antibody and detected with goat anti‐rabbit IgG‐HRP conjugate as described in Experimental Procedures. Bars represent standard error of duplicate serum samples diluted (1/400). PS 151 reacted with sera raised against all studied *Lactobacillus* strains, whereas PS 142 reacted only with serum against cells of *L. johnsonii* 142. Neither GL1 nor GL2 showed reactivity with tested sera, with an exception of sera against LJ 151 and LR 115. Sera from non‐immunized animals were used as controls and presented on Fig. S3.

Immunological activity of glycoconjugates isolated from *L. johnsonii* (LJ142 and LJ151), both glycolipids and polysaccharides (Górska *et al*., 2010; Górska‐Frączek *et al*., 2013), was tested by ELISA using sera from rabbits immunized with whole cells of LJ 151 and 142, *L. animalis/murinus* 148, *L. reuteri* 115 and *L. casei* 0912 (Fig. 2). Pure polysaccharide LJ 151 reacted with sera against cells of all studied *Lactobacillus* strains, whereas PS 142 reacted only with serum against cells of LJ 142. In the case of the glycolipids, neither GL1 nor GL2 showed reactivity with tested sera, with an exception of sera against LJ 151 and LR 115. Very weak reactivity, although with the antibody titre consistently higher for GL1 than for GL2, was observed.

### Immunoreactivity of glycolipids and polysaccharides with IBD patients' sera

**Figure 3:**
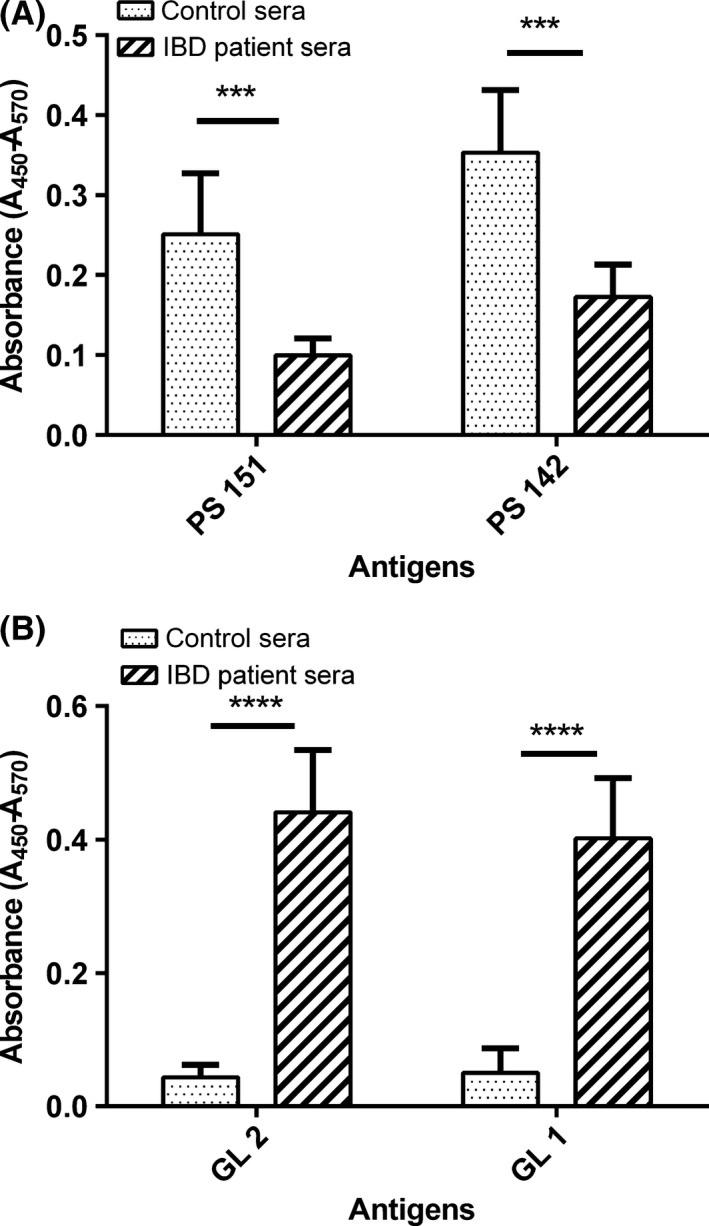
Reactivity in ELISA of *L. johnsonii* polysaccharides and glycolipids with sera of IBD patients and healthy volunteers. Ten sera of the IBD patients were used: three patients with active ulcerative colitis, three patients were with active Crohn's disease and four with inactive ulcerative colitis and the control sera from healthy blood donors (*n* = 10). ELISA plates were coated with polysaccharides (PS 142, PS 151) (A) and glycolipids (GL1, GL2) (B) from *L. johnsonii* strains 151 and 142, and all sera were diluted (1/400). Assays were performed in duplicates, and the mean ± SE is indicated. Statistical significance was assessed by performing two‐way analysis of variance. *P*‐values are indicated (***P < 0.001, ****P < 0.0001). All sera showed the presence of antibodies recognizing both glycolipids and polysaccharides, but at varying titres. Sera of the control group showed higher reactivity with PS 151, PS 142 than with glycolipids, while the sera from the IBD patients reacted better with glycolipids than with polysaccharides.

The reactivity of glycolipids and polysaccharides LJ 142 and LJ 151 with human sera obtained from healthy volunteers and patients with IBD have been examined with ELISA (Fig. 3). All sera showed the presence of IgG antibodies recognizing polysaccharides, but at varying titres. Sera of the control group showed higher reactivity with PS 151 and PS 142 than sera of IBD patients. Interestingly, the sera from the IBD patients reacted with glycolipids significantly better than sera from healthy control group (*P* < 0.001).

## Discussion

Bacterial glycolipids are valuable chemical markers characteristic of genus, or species, allowing for identification of the microorganisms (Mordarska, 1985/1986; Paściak *et al*., 2003, 2014). Glycoglycerolipids, especially diglycosyl diglycerides are widespread among Gram‐positive bacteria. For these common compounds, differences in sugars or in fatty acids can be characteristic of the genus, e.g. Glcα1‐2Glcα1‐3'DG is found in *Streptococcus*, Glcβ1‐6Glcβ1‐3'DG in *Staphylococcus* or Galβ1‐2Galβ1‐3'DG in *Bifidobacterium* (Wiegandt, 1985; Iwamori *et al*., 2015).

Described herein glycolipids from *L. johnsoni* 142 and 151 belong to di‐ and tri‐glycosyldiglycerides, while tetraglycosyldiglycerides were not detected in these strains. Despite different sources, the major glycolipids for both strains are quite similar in chemical composition and in structure. Glycolipid GL2, determined as α‐D‐Gal*p*‐(1→2)‐α‐D‐Glc*p*‐diglyceride, could potentially serve as taxonomic marker for *Lactobacillus,* according to our results (Fig. S1) and the results obtained by Iwamori group (Iwamori *et al*., 2011, 2015). Glycolipid GL1 of *L. johnsonii*, β‐D‐Glc*p*‐(1→6)‐α‐D‐Gal*p*‐(1→2)‐α‐D‐Glc*p*‐diglyceride, is structurally similar to glycolipid GL3 of *L. plantarum* but possess different length of fatty acids, i.e. shorter, palmitic and lactobacillic acids instead of oleic and dihydrosterulic (9,10‐methyleneoctadecanoic) acids (Sauvageau *et al*., 2012). In LJ Galα1‐6Galα1‐6Galα1‐2Glcα1‐3'DG as major tetraglycosyldiglyceride was reported previously, and also Galα1‐6Galα1‐2Glcα1‐3'DG and Galα1‐2Glcα1‐3'DG (Iwamori *et al*., 2011). The differences in glycolipid structures found in the same species arise mostly from different strains studied.

As we have shown, polyclonal antibodies against polysaccharides and glycolipids can be produced after immunization of rabbits with whole lactobacilli cells. No differences were observed in both glycolipids reactivity with antibodies raised against strains LJ 142 and 151, as well as glycolipids isolated from two different strains (Fig. 2). Unlike glycolipids, the anti‐polysaccharide antibodies showed some divergence. The PS 142 reactivity was limited to homologous serum, while PS 151 expressed broad serological cross‐reactivity. These results were in agreement with previously published data (Górska *et al*., 2010; Górska‐Frączek *et al*., 2013). Interestingly, we also observed that only polysaccharides and not glycolipids reacted with mouse non‐immune sera (Fig. S3).

It was interesting to find out if such antibodies are also present in human sera. Studies conducted by Iwamori group revealed that human sera contain antibodies towards lactobacilli glycoglycerolipids, but with titres lacking consistency (Iwamori *et al*., 2011). The antibody titre to tetraglycosyldiglycerides in individual sera was constantly higher than those to diglycosyldiglycerides and triglycosyldiglycerides, irrespective of the blood group. It is worth to note that antibodies against Glcβ1‐6Galα1‐2Glcα1‐3'DG were detected also in human sera but at a marginal level (Iwamori *et al*., 2015). However, the anti‐*L. johnsonii* sera reacted with Glcβ1‐6Galα1‐2Glcα‐diglyceride at 40% of the activity determined for Galα1‐6Galα1‐6Galα1‐2Glcα1‐3'DG (Iwamori *et al*., 2015).

For the purpose of this study, we have chosen sera from patients with recognized IBD. We were prompted by the literature reports suggesting bacterial aetiology of this disease (Sartor and Mazmanian, 2012), and because one of the studied strains (LJ 142) was obtained from mouse with experimentally induced IBD.

Our results show that immunological response to commensal bacteria in IBD patients is different than that in healthy individuals. Interestingly, sera of the IBD patients showed a low level of anti‐PS antibodies and statistically significant higher level of anti‐glycolipid antibodies. This finding suggests a breakdown in tolerance to normal commensal microbiota of the intestine. IgGs could also be directed against other antigens, such as epithelial glycolipids, which could have been discharged during inflammation process. The perturbations can affect different level of the protective mechanisms, including alterations to a pattern recognition receptors. Iwamori group (Iwamori *et al*., 2013) demonstrated that glycolipids of the receptors active towards bacteria in the digestive tract were metabolized in response to alteration in the intestinal bacterial population.

In contrast to healthy individuals, sera from the IBD patients produce a high level of IgG resulting from the antigen‐specific activation of the lymphocytes within the intestinal mucosa. However, these commensal microbiota antigens against which these mucosal immunoglobulins are directed have received relatively little attention (Scott *et al*., 1986). The sera of the IBD patients studied so far showed a high antibody titre against cytoplasmic proteins from both Gram‐positive and Gram‐negative commensal bacteria (Macpherson *et al*., 1996). Apart from antibodies directed against the cytoplasmic proteins, the ones towards lipid A (Oriishi *et al*., 1995) and glycans (Dotan *et al*., 2006) were also found.

In IBD patients’ sera apart from antibodies to self, those against bacterial and fungal antigens have also been observed. These antibodies have only a limited role as diagnostic serologic markers for IBD, mainly because of their low sensitivity (Vermeulen *et al*., 2008). New biomarkers for all aspects of the IBD patients’ clinical care are needed (Iskandar and Ciorba, 2012). The LJ cell wall glycoconjugates, i.e. glycolipids and polysaccharides, have a potential to become biomarkers for evaluating the immune status of the patient.

The weakening of the host defence mechanism may allow for more bacteria to come in a direct contact with the epithelium and mucosal immune cells, and consequently trigger compensatory immune reaction. The anti‐glycolipids antibodies would appear in human sera as a result of an immune reaction against these bacteria, probably to protect the host. Iwamori *et al*. (2011) studied structure of the glycolipids from different strains of *L. johnsonii* and found anti‐glycolipid IgM antibodies at various titres in human sera, indicating that an immune reactivity to symbiotic lactobacilli occurs mainly against trihexaosyl diacylglycerol (TH‐DG) and tetrahexaosyl diacylglycerol (TetH‐DG). Furthermore, the antibodies towards TH‐DG and TetH‐DG in symbiotic lactobacilli have not reacted with normal tissues or cells of the host. However, during infection with pathogen *Campylobacter jejuni*, antibodies against oligosaccharides mimicking host's epithelial gangliosides, were found to be produced (Iwamori *et al*., 2011).

To sum up, the major glycolipids of LJ 142 and 151 have been identified as β‐D‐Glc*p*‐(1→6)‐α‐D‐Gal*p*‐(1→2)‐α‐D‐Glc*p*‐diglyceride (GL1) and α‐D‐Gal*p*‐(1→2)‐α‐D‐Glc*p*‐diglyceride (GL2). Glycolipid GL2 possesses taxonomic value and could be taxonomic marker in *Lactobacillus* genus. Results of immunoreactivity of *L. johnsonii* glycolipids and polysaccharides with sera from IBD patients open a possibility for using these antigens in the development of a diagnostic test. Although promising, more sera from IBD patients and healthy individuals are needed to validate this premise.

## Experimental procedures

### Bacteria

The LJ strain 151 (LJ 151) isolated from murine gastrointestinal tract and strain of LJ 142 from an intestinal tract of mice with experimentally induced IBD (Górska *et al*., 2010) both isolated and deposited in Collegium Medicum of Jagiellonian University in Krakow, were used throughout the studies. The CD4^+^ CD45RB high T‐cell transfer SCID mice were used as an animal model of IBD (Morrissey *et al*., 1993; Powrie *et al*., 1993). Other strains used for comparison, namely *Lactobacillus reuteri* strains 130 (LR 130) and 115 (LR 115), *L. casei* 0912 and PCM 2639, *L. animalis/murinus* 148 (LAM 148) and *L. rhamnosus* PCM 492, were from Collegium Medicum and Polish Collection of Microorganisms (PCM) respectively. The lactobacilli were cultivated on MRS broth for 72 h at 37°C under anaerobic conditions. Bacteria were harvested by centrifugation (6000 r.p.m., 4°C, 20 min) and cells were washed thrice with PBS and water.

### Crude lipid preparation and analysis

The wet cell mass (10 g) samples of the LJ 142 and 151 were treated twice with chloroform/methanol (2:1, v/v, 150 ml) at 37°C for 12 h. The crude lipid samples of *L. reuteri*,* L. casei*,* L. animalis/murinus* and *L. rhamnosus* were obtained from smaller amount of wet cell mass (1 g) after 12 h extraction with chloroform–methanol mixture (2:1 v/v) at 37°C. The lipid content was monitored by TLC on silica gel plates run in solvent system composed of chloroform–methanol–water (65:25:4 v/v/v) and stained with orcinol (Mordarska and Paściak, 1994). Chromatograms were also stained with vanillin, ninhydrin and molybdate reagent specific for phosphorus (Dittmer and Lester, 1964).

### Purification of glycolipids by chromatographic methods

The crude lipid extract (about 500 mg) was loaded on a column (1.8 × 60 cm) of activated (at 120°C) Silica Gel 60 (230‐400 mesh ASTM; Merck, Warszawa, Poland) and eluted successively with chloroform, acetone and methanol (4 × 100 ml of each solvent). The eluates were monitored for polar lipids by TLC. The acetone fractions that contained glycolipids were further fractionated on a column (1.5 × 45 cm) of Silica Gel 60 (200–300 mesh; Merck) with a step‐gradient of MeOH in chloroform, (0, 5, 10, 15, 20, 30 and 50 vol %) and at the flow rate of 1 ml min^−1^. Fractions of 50 ml were collected and lipids were monitored by TLC as before. Fractions that contained similarly migrating glycolipids were combined and further purified by HPLC.

The final purification step was performed by using HPLC (Waters 996; Millipore, Milford, MA, USA) equipped with an M600 pump and Photodiode Array Detector. The HPLC system was controlled and chromatographic data collected with Millennium 2010 software (Waters, Millipore, Milford, MA, USA). The glycolipids GL1 and GL2 were purified using a Silica Gel column PARTISIL 5 (PARTISIL Si 5 μm 25 × 0.46 cm; Teknokroma, Barcelona, Spain) and gradient of MeOH in chloroform (from 15% to 25%) with flow rate 1 ml min^−1^. Fractions were collected every 1 min, over 30 min, and purity of the samples was monitored by TLC.

### Chemical analysis

The total sugar content was determined by phenol–sulfuric acid method using glucose (0–50 μg) as a standard (Dubois *et al*., 1956).

The neutral sugar composition was established by acid hydrolysis of glycolipids, derivatization of the monosaccharides to alditol acetate and analysis by GLC‐MS (Sawardeker *et al*., 1965). Briefly, samples were hydrolysed with 2 M trifluoroacetic acid (120°C, 2 h), followed by evaporation under a stream of nitrogen, extracted with chloroform–water followed by reduction with NaBH_4_ and acetylation with acetic anhydride in pyridine. The alditol acetates were detected by GLC‐MS. Thermo Scientific ITQ 700 Focus GC equipped with a Rxi‐5ms (Restek, Bellefonte, PA, USA) capillary column (30 m × 0.25 mm) was used. Derivatized sugars were resolved by applying temperature gradient of 150–260°C at 8°C min^−1^.

Methylation of glycolipid samples (0.2 mg) and their analysis was performed as described by Ciucanu and Kerek (1984). After extraction into chloroform, the methylated glycolipids were hydrolysed with 4M TFA, reduced with NaBD_4_ and acetylated as described above. Trace GC ultra (Thermo Scientific. Waltham, MA, USA) TSQ Quantum instrument equipped with a Zebron ZB‐5MS w/5 Meter GUARDIAN (30 m × 0.25 mm, 0.25 μm) capillary column (Phenomenex, Torrance, CA, USA) and a temperature gradient of 150–270°C at 12°C min^−1^, was used for the analysis. Peak retention times and mass spectra analysis were used to identify methylated sugars.

For fatty acid analysis, 0.1 mg of purified glycolipids were treated with 1 ml of methanol–chloroform–hydrochloric acid (10:1:1, v/v/v) and heated at 80°C for 1 h (Nichols *et al*., 2002). After adding 1 ml of Milli‐Q water to each sample, the fatty acid methyl esters were extracted three times with 1.5 ml of hexane–chloroform (4:1, v/v) and analysed by GLC‐MS (Thermo Scientific ITQ 700 Focus GC).

### NMR analysis

Purified glycolipids were dissolved in CDCl_3_/CD_3_OD (2:1, v/v). Samples were analysed at 22°C and the chemical shift measured relative to chloroform. The NMR spectra were obtained on a Bruker 600 MHz Avance III spectroscope using a micro TXI probe (Bruker, BioSpin, Fällanden, Switzerland). The data were acquired and processed using Bruker Topspin software (version 3.1). The signals were assigned using 1D and 2D experiments, COSY, TOCSY, ROESY, HSQC and HMBC. The TOCSY experiments were carried out with mixing times of 30, 60 and 100 ms.

### MALDI‐TOF analysis

MALDI‐TOF MS was performed in the positive ion sweep, linear and reflectron mode, using the Ultraflextreme unit (Bruker Daltonics, Germany). The samples were dissolved in chloroform–MeOH (9:1 v/v) at a concentration of 1 mg ml^−1^. Matrix solution was prepared from norharmane (Sigma‐Aldrich, Poznan, Poland) in chloroform–MeOH (9:1, v/v) at a concentration of 5 mg ml^−1^. Matrix and sample solution were mixed and applied onto the stainless steel target plate as 1.0 μl droplets. Samples were allowed to crystallize at RT.

Tandem mass spectra were recorded using the Ultraflextreme unit with LIFT technology (Suckau *et al*., 2003). The accelerating voltages were 7.50 kV and 6.75 kV for ion sources 1 and 2 respectively. The reflector 1 and 2 were set to 29.5 kV and 13.95 kV respectively, with Lift 1 and 2 set at 19 kV and 3.35 kV. The precursor ion selector was set manually to the first monoisotopic peak of the molecular ion pattern for all analyses. The MS/MS spectra were acquired from 2500 laser shots for each glycolipid. Spectroscopic data were analysed with Bruker Daltonics Flex Analysis Software (version 3.4).

### Rabbit immune sera

Rabbits were immunized with LJ 151, LJ 142, *L. animalis/murinus* 148, *L. reuteri* 115 and *L. casei* 0912 cells suspended in PBS and sera were obtained as described (Górska‐Frączek *et al*., 2013) with one exception. In case of the LJ 151 sample, bacteria were homogenized by ultrasonication before they were used for immunization. The experiments were approved by the 1^st^ Local Committee for Experiments with the Use of Laboratory Animals, Wroclaw, Poland.

The antisera were tested by conventional passive hemagglutination test (Penner and Hennessy, 1980). Double immunodiffusion test was carried out on plates coated with 1% agarose in PBS (Ouchterlony, 1948). The central well was filled with LJ 151 cell mass suspension (1 mg ml^−1^), and external wells with rabbit serum towards LJ 142 and 151 undiluted and two times diluted. Plate was examined after 24, 48 and 72 h of incubation at 4°C.

### Mouse sera

Non‐immune mouse sera were obtained from 6‐ to 7‐week‐old mice of the inbred 129/Ao/Boy/IiW strain of both sexes. Mice weighing approximately 20 g were obtained from the Breeding Unit of the Medical University of Wroclaw, Poland. Animals were held under specific pathogen‐free conditions. Mice were bled, the separated antisera were decomplemented (56°C, 30 min) and stored at −20°C. The experiments were approved by the 1st Local Committee for Experiments with the Use of Laboratory Animals, Wroclaw, Poland.

### Human sera

For the pilot ELISA study, 10 sera of the IBD patients (six males and four females) were used: three patients had active ulcerative colitis, three patients were with active Crohn's disease and four with inactive ulcerative colitis (mean age 40.9, range 25–66). Sera were obtained from the Department of Medical Biochemistry of Wroclaw Medical University (Matusiewicz *et al*., 2014). The control sera (*n* = 10) were obtained from healthy blood donors. The study protocol was approved by the Medical Ethics Committee of Wroclaw Medical University, Wroclaw, Poland and the study was conducted in accordance with the Helsinki Declaration of 1975, as revised in 1983.

### Enzyme‐linked immunosorbent assay

The direct ELISA experiments were performed with glycolipids (GL1, GL2) and polysaccharides isolated from LJ 151 (PS 151) (Górska‐Frączek *et al*., 2013) and LJ 142 (PS 142) (Górska *et al*., 2010) adsorbed to the flat‐bottom 96‐well plates. MaxiSorp plates (Thermo Fisher Scientific, Roskilde, Denmark) were coated overnight at 4°C with 100 μl of a 0.01 mg ml^−1^ polysaccharide solution, while for glycolipids, polyvinyl chloride plates (Falcon, Becton Dickinson, Oxnard, CA, USA) were coated with GL1 and GL2 (250 ng/50 μl) dissolved in methanol, dried at room temperature and stored overnight in desiccator at 4°C. Plates were washed three times with PBS‐T (PBS + 0.05% Tween 20) and blocked at 25°C for 1 h with 0.2% casein in PBS (w/v). Plates were again washed 3 times with PBS‐T and incubated with 100 μl of rabbit serum (geometric dilution from 1:3200 to 1:51 200 in PBS), mouse serum (geometric dilution from 1:400 to 1:6400 in PBS) or sera from human subjects (IBD patients and control blood donors) diluted with PBS from 1:400 to 1:6400 in PBS, at 37°C for 2 h. Following five PBS‐T washes, the horseradish peroxidase‐conjugated goat anti‐rabbit IgG (1:2000; DakoCytomation, Glostrup, Denmark), goat anti‐mouse IgG (1:2000; DakoCytomation, Glostrup, Denmark) and goat anti‐human IgG (1:10 000; Sigma, Fc‐specific) were applied and the plates were incubated at 25°C for 1 h. After five washing steps with PBS‐T, 100 μl of 3,3′,5,5′‐tetramethylbenzidine (TMB; Sigma) peroxidase substrate was applied and incubated at 25°C for 30 min. Enzymatic reaction was stopped with 50 μl of 2M H_2_SO_4_ and absorbance read at 450 and 570 nm on the plate‐reader (BioTek, Winooski, VT, USA).

### Statistical analysis

Data are expressed as means and standard errors of the means (SEM). Statistical analysis was performed by two‐way ANOVA test using Prism 5.06 software (GraphPad, San Diego, CA, USA). *P* < 0.05 was considered significant.

## Conflict of interest

The authors declare no conflict of interest.

## Supporting information


**Fig. S1.** Glycolipid patterns of *Lactobacillus* strains. TLC chromatogram of glycolipids from: 1. *L. johnsonii* 142, 2. *L. johnsonii* 151, 3. *L. reuteri* 130, 4*. L. casei* PCM 2639, 5. *L. reuteri* 115, 6. *L. animalis*/*murinus* 148, 7. *L. rhamnosus* PCM 492. Solvent system: chloroform–methanol–water (65:25:4, v/v/v), detection: orcinol reagent (Mordarska and Paściak, 1994). Glycolipid GL2 is a major glycolipid of *Lactobacillus* genus.
**Fig. S2.** Parts of ^1^H NMR spectra of glycolipids GL2 from *L. johnsonii* 142 (A) and 151 (B) strains. The spectra were obtained for CDCl_3_/CD_3_OD (2:1, v/v) solvent at 600 MHz and 22°C. The letters refer to carbohydrate residues and the Arabic numerals refer to proton in the respective residue denoted as shown in Table 2. Structures of GL2 in both strains are identical.
**Fig. S3.** Reactivity in ELISA of *L. johnsonii* polysaccharides and glycolipids with rabbit and mouse non‐immune sera. Rabbit sera taken before immunization with cell mass of *L. johnsonii* 142 (LJ142) and *L. johnsonii* 151 (LJ151) and mouse non‐immune sera were used as the primary antibody and detected with goat anti‐rabbit IgG‐HRP conjugate and goat anti‐mouse IgG‐HRP conjugate respectively. The ELISA plates were coated with polysaccharides (PS 142, PS 151) (A) and glycolipids (GL1, GL2) (B) from *L. johnsonii* strains 151 and 142 and all sera were diluted 400x. Bars represent standard error of duplicate serum samples. Rabbits’ sera before immunization had no reactivity with polysaccharides and glycolipids *of L. johnsonii*, whereas mice non‐immune sera reacted with polysaccharide 151 and 142.
**Table S1.** ^1^H and ^13^C NMR chemical shifts of the fatty acid alkyl signals from GL1 and GL2 of *L. johnsonii* 151. Proton signals were assigned in the COSY, TOCSY and HMBC spectra, whereas carbon signals were assigned in the HSQC spectrum. Spectra were obtained for CDCl_3_/CD_3_OD (2:1, v/v) solvent at 22°C and the chemical shifts measured relative to chloroform.
