# Supplementary material for: Lactobacillus johnsonii glycolipids, their structure and immunoreactivity with sera from inflammatory bowel disease patients
Source: Microb Biotechnol. 2016 Oct 21;10(2):456–68. doi: 10.1111/1751-7915.12424 (PMC5328823; doi:10.1111/1751-7915.12424)
Supplement: Supplementary file 1 — Fig. S1. Glycolipid patterns of Lactobacillus strains. TLC chromatogram of glycolipids from: 1. L. johnsonii 142, 2. L. johnsonii 151, 3. L. reuteri 130, 4. L. casei PCM 2639, 5. L. reuteri 115, 6. L. animalis/murinus 148, 7. L. rhamnosus PCM 492. Solvent system: chloroform–methanol–water (65:25:4, v/v/v), detection: orcinol reagent (Mordarska and Paściak, 1994). Glycolipid GL2 is a major glycolipid of Lactobacillus genus. Fig. S2. Parts of 1H NMR spectra of glycolipids GL2 from L. johnsonii 142 (A) and 151 (B) strains. The spectra were obtained for CDCl3/CD3OD (2:1, v/v) solvent at 600 MHz and 22°C. The letters refer to carbohydrate residues and the Arabic numerals refer to proton in the respective residue denoted as shown in Table 2. Structures of GL2 in both strains are identical. Fig. S3. Reactivity in ELISA of L. johnsonii polysaccharides and glycolipids with rabbit and mouse non‐immune sera. Rabbit sera taken before immunization with cell mass of L. johnsonii 142 (LJ142) and L. johnsonii 151 (LJ151) and mouse non‐immune sera were used as the primary antibody and detected with goat anti‐rabbit IgG‐HRP conjugate and goat anti‐mouse IgG‐HRP conjugate respectively. The ELISA plates were coated with polysaccharides (PS 142, PS 151) (A) and glycolipids (GL1, GL2) (B) from L. johnsonii strains 151 and 142 and all sera were diluted 400x. Bars represent standard error of duplicate serum samples. Rabbits’ sera before immunization had no reactivity with polysaccharides and glycolipids of L. johnsonii, whereas mice non‐immune sera reacted with polysaccharide 151 and 142. Table S1. 1H and 13C NMR chemical shifts of the fatty acid alkyl signals from GL1 and GL2 of L. johnsonii 151. Proton signals were assigned in the COSY, TOCSY and HMBC spectra, whereas carbon signals were assigned in the HSQC spectrum. Spectra were obtained for CDCl3/CD3OD (2:1, v/v) solvent at 22°C and the chemical shifts measured relative to chloroform. [file MBT2-10-456-s001.docx]

**Description**

**Fig. S1. Glycolipid patterns of *Lactobacillus* strains.** TLC chromatogram of glycolipids from: 1. *L. johnsonii* 142, 2. *L. johnsonii* 151, 3. *L. reuteri* 130, 4. *L. casei* PCM 2639, 5. *L. reuteri* 115, 6. *L. animalis/murinus* 148, 7. *L. rhamnosus* PCM 492. Solvent system: chloroform-methanol-water (65:25:4, v/v/v), detection: orcinol reagent (Mordarska and Paściak, 1994). Glycolipid GL2 is a major glycolipid of *Lactobacillus* genus.

**Fig. S2. Parts of ^1^H NMR spectra of glycolipids GL2 from *L. johnsonii* 142 (A) and 151 (B) strains.** The spectra were obtained for CDCl_3_/CD_3_OD (2:1, v/v) solvent at 600 MHz and 22 °C. The letters refer to carbohydrate residues and the Arabic numerals refer to proton in the respective residue denoted as shown in Table 2. Structures of GL2 in both strains are identical.

**Fig. S3. Reactivity in ELISA of *L. johnsonii* polysaccharides and glycolipids with rabbit and mouse non-immune sera**. Rabbit sera taken before immunization with cell mass of *L. johnsonii* 142 (LJ142) and *L. johnsonii* 151 (LJ151) and mouse non-immune sera were used as the primary antibody and detected with goat-anti-rabbit IgG-HRP conjugate and goat-anti-mouse IgG-HRP conjugate, respectively. The ELISA plates were coated with polysaccharides (PS 142, PS 151) (A) and glycolipids (GL1, GL2) (B) from *L. johnsonii* strains 151 and 142 and all sera were diluted 400x. Bars represent standard error of duplicate serum samples. Rabbits sera before immunization had no reactivity with polysaccharides and glycolipids of *L. johnsonii*, whereas mice non-immune sera reacted with polysaccharide 151 and 142.

**Table S1**. **^1^H and ^13^C NMR chemical shifts of the fatty acid alkyl signals from GL1 and GL2 of *L. johnsonii* 151.** Proton signals were assigned in the COSY, TOCSY and HMBC spectra, whereas carbon signals were assigned in the HSQC spectrum. Spectra were obtained for CDCl_3_/CD_3_OD (2:1, v/v) solvent at 22 °C and the chemical shifts measured relative to chloroform.


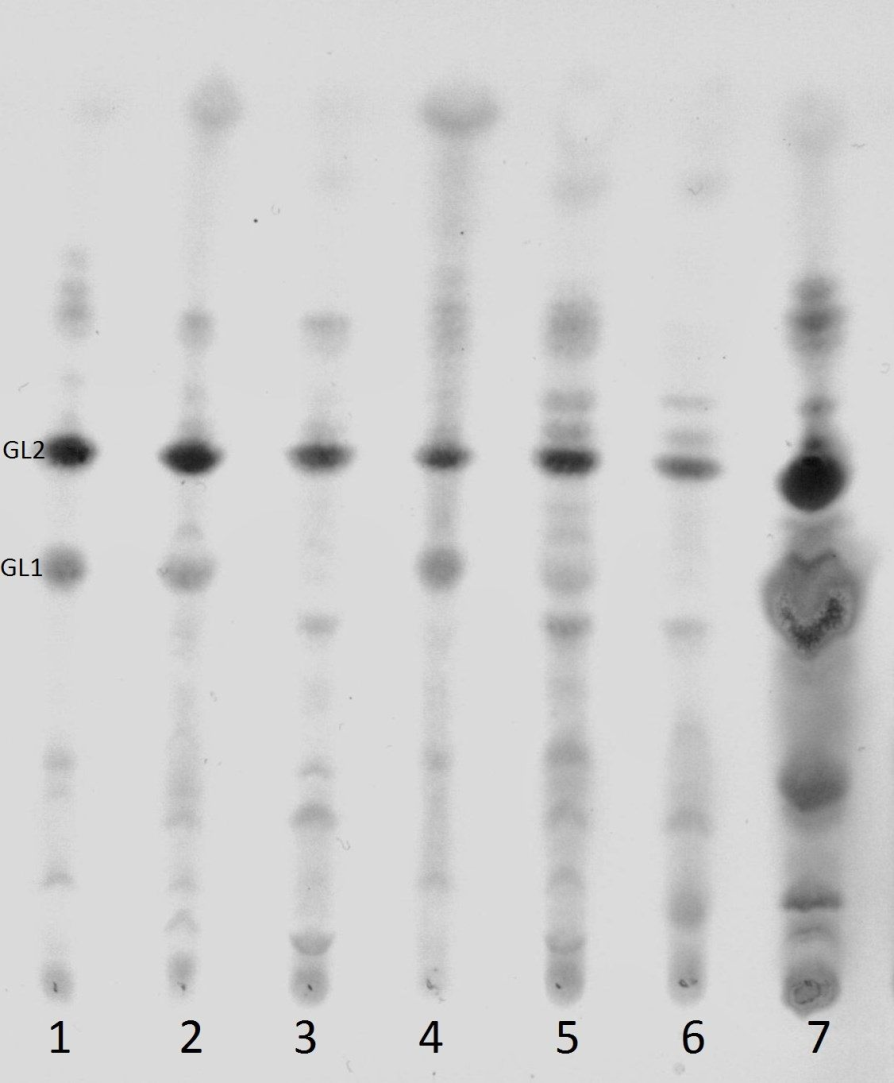


**Fig. S1. Glycolipid patterns of *Lactobacillus* strains.** TLC chromatogram of glycolipids from: 1. *L. johnsonii* 142, 2. *L. johnsonii* 151, 3. *L. reuteri* 130, 4. *L. casei* PCM 2639, 5. *L. reuteri* 115, 6. *L. animalis/murinus* 148, 7. *L. rhamnosus* PCM 492. Solvent system: chloroform-methanol-water (65:25:4, v/v/v), detection: orcinol reagent (Mordarska and Paściak, 1994). Glycolipid GL2 is a major glycolipid of *Lactobacillus* genus.





**Fig. S2. Parts of ^1^H NMR spectra of glycolipids GL2 from *L. johnsonii* 142 (A) and 151 (B) strains.** The spectra were obtained for CDCl_3_/CD_3_OD (2:1, v/v) solvent at 600 MHz and 22 °C. The letters refer to carbohydrate residues and the Arabic numerals refer to proton in the respective residue denoted as shown in Table 2. Structures of GL2 in both strains are identical.





**Fig. S3. Reactivity in ELISA of *L. johnsonii* polysaccharides and glycolipids with rabbit and mouse non-immune sera.** Rabbit sera taken before immunization with cell mass of *L. johnsonii* 142 (LJ142) and *L. johnsonii* 151 (LJ151) and mouse non-immune sera were used as the primary antibody and detected with goat-anti-rabbit IgG-HRP conjugate and goat-anti-mouse IgG-HRP conjugate, respectively. The ELISA plates were coated with polysaccharides (PS 142, PS 151) (A) and glycolipids (GL1, GL2) (B) from *L. johnsonii* strains 151 and 142 and all sera were diluted 400x. Bars represent standard error of duplicate serum samples. Rabbits sera before immunization had no reactivity with polysaccharides and glycolipids of *L. johnsonii*, whereas mice non-immune sera reacted with polysaccharide 151 and 142.

**Table S1**. ^1^H and ^13^C NMR chemical shifts of the fatty acid alkyl signals from GL1 and GL2 of *L*. *johnsonii* 151. Proton signals were assigned in the COSY, TOCSY and HMBC spectra, whereas carbon signals were assigned in the HSQC spectrum. Spectra were obtained for CDCl_3_/CD_3_OD (2:1, v/v) solvent at 22 °C and the chemical shifts measured relative to chloroform.

| **Fatty acid residue** | **Group** | **Chemical shifts ^1^H and ^13^C [*δ*]** | |
| --- | --- | --- | --- |
|  |  | **Protons shifts** | **Carbon shifts** |
| R1 | 1-C | — | 174.4 |
|  | 2-CH_2_ | 2.33 | 34.5 |
|  | 3-CH_2_ | 1.62 | 24.6 |
|  | n-CH_2_ | 1.25-1.35 | 29.5-31.5 |
|  | *n*-CH_3_ | 0.89 | 14.2 |
|  | –C*H_2_*–CH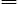 | 2.026 | 27.7 |
|  | -CH_2_–C*H*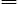 | 5.358 | 129.9 |
|  | | | |
| R2 | 1-C | — | 174.6 |
|  | 2-CH_2_ | 2.33 | 34.5 |
|  | 3-CH_2_ | 1.622 | 24.6 |
|  | n-CH_2_ | 1.15-1.35 | 28.5-32.0 |
|  | n-CH | 0.66 | 16.0 |
|  | *n*-CH_3_ | 0.89 | 14.2 |
|  |  |  |  |

*n*—number of carbon atoms in a fatty acid chain.
